# Biliary ascariasis: extraction

**DOI:** 10.1055/a-2258-8436

**Published:** 2024-02-22

**Authors:** Muhammad Rahimi, Ikhwan Sani Mohamad, Maya Mazuwin Yahya, Mohd Azem Fathi Mohammad Azmi, Leow Voon Meng

**Affiliations:** 1 Department of Surgery, Universiti Sains Malaysia, Kubang Kerian, Malaysia; 2 Hospital Universiti Sains Malaysia, Kubang Kerian, Malaysia; 3 Advanced Medical and Dental Institute, Universiti Sains Malaysia, Kepala Batas, Malaysia

Biliary ascariasis is one of the complications of *Ascaris lumbricoides* infestation. The condition results from the migration of the *A. lumbricoides* worm into the biliary tract, causing symptoms ranging from mild biliary colic to severe cholangitis 1 . Despite *A. lumbricoides* being one of the most common helminth infestations in Malaysia, biliary ascariasis is rare 2 .

A 71-year-old woman with a history of laparoscopic cholecystectomy presented with sudden onset of abdominal pain localized at the epigastric region and radiating to the back. She had episodes of low grade fever and a history of passing out worms in stool a day prior to presentation to our center. The patient was from a poor socioeconomic background, with constant exposure to soil and farm animals.

Ultrasound assessment showed a dilated common hepatic duct of 1.4 cm in diameter, with the presence of a linear tubular echogenic structure within the biliary tree ( Fig. 1 ). Fluoroscopic imaging showed a moving elongated structure within the biliary tree ( Fig. 2 ).

**Fig. 1:**
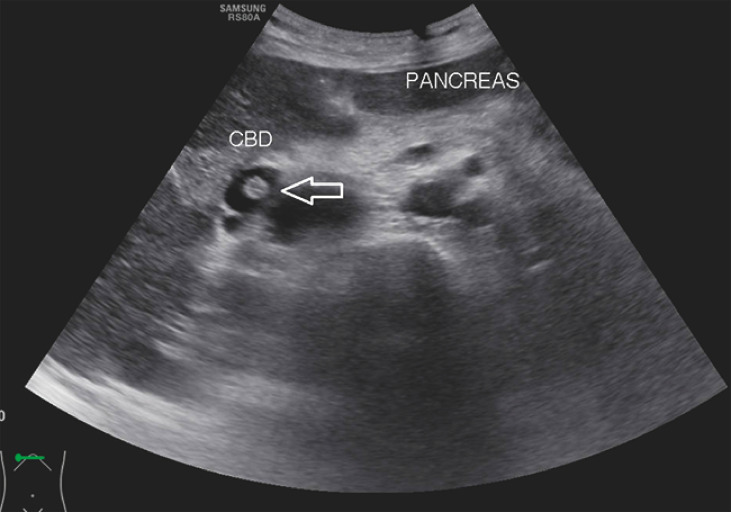
Ultrasonography showed the presence of a worm (arrow) within the biliary tree.

**Fig. 2:**
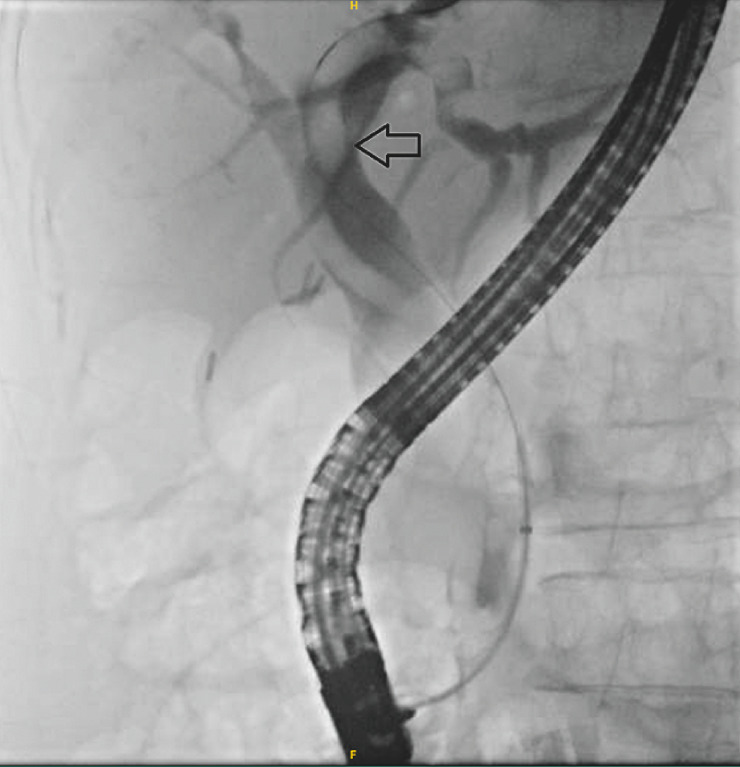
Fluoroscopy demonstrated the presence of *Ascaris Lumbricoides* (arrow).

Endoscopic retrograde cholangiopancreatography (ERCP) was performed and the solitary worm was retrieved via the oral route by using balloon trawl and snare probe. The worm was then inspected and measured. The whole worm measured 25 cm in length and was extracted in one piece ( Video 1 ).


Endoscopic retrograde cholangiopancreatography for removal of *Ascaris lumbricoides* . By using the constant radial expansion balloon, the distal common bile duct was dilated and the whole worm was extracted in one piece.
Video 1

The patient was further treated with antihelminth (oral albendazole 400 mg once daily) for 3 days, and was discharged after 3 days of admission.

Biliary ascariasis can be diagnosed by noninvasive imaging such as ultrasonography as the primary investigation tool 3 . It can be further confirmed and managed by ERCP. Ascariasis in general as a disease is much more difficult to eradicate as its epidemiology involves those who are from poor socioeconomic backgrounds 4 . Without a holistic approach with multidisciplinary involvement at healthcare and community levels, the cycle of ascariasis infestation will remain unbroken.

Endoscopy_UCTN_Code_CCL_1AZ_2AN

## Notes

Endoscopy E-Videos https://eref.thieme.de/e-videos
*E-Videos* is an open access online section of the journal *Endoscopy* , reporting on interesting cases and new techniques in gastroenterological endoscopy. All papers include a high-quality video and are published with a Creative Commons CC-BY license. Endoscopy E-Videos qualify for HINARI discounts and waivers and eligibility is automatically checked during the submission process. We grant 100% waivers to articles whose corresponding authors are based in Group A countries and 50% waivers to those who are based in Group B countries as classified by Research4Life (see: https://www.research4life.org/access/eligibility/ ). This section has its own submission website at https://mc.manuscriptcentral.com/e-videos .
